# Nivolumab plus ipilimumab versus pembrolizumab as chemotherapy‐free, first‐line treatment for PD‐L1‐positive non‐small cell lung cancer

**DOI:** 10.1002/ctm2.14

**Published:** 2020-04-07

**Authors:** Yixin Zhou, Yaqiong Zhang, Guifang Guo, Xiuyu Cai, Hui Yu, Yanyu Cai, Bei Zhang, Shaodong Hong, Li Zhang

**Affiliations:** ^1^ State Key Laboratory of Oncology in South China Guangzhou China; ^2^ Collaborative Innovation Center for Cancer Medicine Guangzhou China; ^3^ Department of VIP region Sun Yat‐sen University Cancer Center Guangzhou China; ^4^ Department of Radiotherapy Sun Yat‐sen University Cancer Center Guangzhou China; ^5^ Department of Medical Oncology Sun Yat‐sen University Cancer Center Guangzhou China

**Keywords:** ipilimumab, nivolumab, non‐small cell lung cancer, pembrolizumab, programmed cell death‐ligand 1

## Abstract

**Background:**

Nivolumab plus ipilimumab (N‐I) or pembrolizumab (PEM) is associated with survival improvement as chemotherapy‐free, first‐line treatment for patients with advanced non‐small cell lung carcinoma (NSCLC) and positive programmed cell death ligand 1 (PD‐L1). However, no direct comparison data exist between these two regimens to inform clinical decisions. Therefore, we performed indirect comparison for N‐I versus PEM using frequentist methods.

**Results:**

Three randomized trials (KEYNOTE‐024, KEYNOTE‐042, and CheckMate 227) involving 2372 patients were included. For patients with tumor PD‐L1 level of ≥1%, pooled meta‐analyses showed that both N‐I and PEM improved overall survival (OS) relative to chemotherapy (N‐I: hazard ratio [HR] 0.82, 95% CI 0.69‐0.97; PEM: HR 0.81, 95% CI 0.71‐0.93); whereas only N‐I significantly improved progression‐free survival (PFS) (N‐I: HR 0.79, 95% CI 0.65‐0.96; PEM: HR 1.07, 95% CI 0.94‐1.21). Neither N‐I nor PEM was associated with improved objective response rate (ORR) compared with chemotherapy (N‐I: relative risk [RR] 1.20, 95% CI 0.98‐1.46; PEM: RR 1.03, 95% CI 0.86‐1.23). Indirect comparisons showed that N‐I was associated with longer PFS than PEM (HR 0.77, 95% CI 0.62‐0.95). However, N‐I was not superior to PEM in terms of OS (HR 0.98, 95% CI 0.77‐1.24) and ORR (RR 1.17, 95% CI 0.89‐1.52). N‐I showed a less favorable toxicity profile relative to PEM (all grade adverse events: RR 1.28, 95% CI 1.17‐1.40).

**Conclusions:**

N‐I and PEM provide comparable OS benefit for PD‐L1‐positive NSCLC. N‐I further improves PFS relative to PEM but at meaningful cost of toxicities.

**Abbreviations**
1L: first‐line
CI: confidence interval
CTLA‐4: cytotoxic T lymphocyte antigen 4
HRs: hazard ratios
N‐I: nivolumab plus ipilimumab
NSCLC: non‐small‐cell lung carcinoma
ORR: objective response rate
OS: overall survival
PD‐1: programmed cell death 1
PD‐L1: programmed cell death ligand 1
PEM: pembrolizumab
PFS: progression‐free survival
RR: relative risk
SE: standard error
TPS: tumor proportion score
TRAEs: treatment‐related adverse events

## 1 INTRODUCTION

For the past two decades, platinum‐based chemotherapy has been the standard‐of‐care, first‐line (1L) treatment for patients with advanced non‐small‐cell lung carcinoma (NSCLC) lacking targetable driver alterations. However, chemotherapy has provided only moderate benefit, with moderate‐to‐severe toxicities.^1^ There exists a great unmet need for more efficacious and tolerable therapy for advanced NSCLC.

Recently, substantial progress has been made in the 1L immunotherapy of advanced NSCLC. These include monotherapy blockade of programmed cell death 1 (PD‐1) in patients with programmed cell death ligand 1 (PD‐L1) tumor proportion score (TPS) of 50% or greater,^2^ or combination with anti‐PD‐(L)1 antibody plus chemotherapy, irrespective of tumor PD‐L1 expression. The KEYNOTE‐042 study further showed that pembrolizumab (PEM) monotherapy provided longer duration of survival than chemotherapy in patients with PD‐L1 TPS of ≥1%.^3^ Still, only a minority of patients obtain long‐term survival. Attempts have been made in simultaneous inhibition of immune checkpoints with complementary mechanisms of action to further improve efficacy. Accordingly, the CheckMate 227 study demonstrated survival improvement with dual inhibition of PD‐1 (nivolumab) and cytotoxic T lymphocyte antigen 4 (CTLA‐4) (ipilimumab) in patients with advanced NSCLC and PD‐L1 TPS of ≥1%, as compared with chemotherapy.^4^ Thus, both nivolumab plus ipilimumab (N‐I) and PEM monotherapy were recommended as chemotherapy‐free 1L treatment for PD‐L1‐positive NSCLC by the recently published National Comprehensive Cancer Network Clinical Practice Guidelines.^5^ However, no direct comparison data exist between these two regimens to be able to make informed patient selection, treatment decisions, and guideline recommendations.

As such, we performed this indirect comparison of efficacy and safety outcomes between N‐I and PEM in advanced NSCLC with established approaches.^6^

## 2 METHODS

### 2.1 Study eligibility

Pubmed, Embase, and the Cochrane Center Register were searched for studies indexed from inception to October 8, 2019 by a professional librarian. We used both subject headings and text‐word terms for “pembrolizumab,” “nivolumab,” “ipilimumab,” “non‐small‐cell lung cancer,” and “randomized controlled trial.” A full search strategy is shown in Supporting Information Methods. We also reviewed the major oncology conference proceedings. Study selection was conducted by two investigators independently, with discrepancy solved by consensus. Only English language publications were considered.

### 2.2 Data extraction

The outcomes of this combined analysis included overall survival (OS), progression‐free survival (PFS), objective response rate (ORR), and treatment‐related adverse events (TRAEs). We derived the hazard ratios (HRs) and its 95% confidence intervals (CIs) for OS and PFS, and the dichotomous data for ORR and TRAEs.

### 2.3 Data analyses

We calculated the pooled HRs, 95% Cis, and *P*‐values for OS and PFS using inverse‐variance‐weighted method. Pooled relative risks (RRs), 95% Cis, and *P*‐values for ORR and TRAEs were computed using the Mantel Haenszel method with a fixed‐effect model. The adjusted indirect comparison was performed on arm A (N‐I) versus arm B (PEM), linked by arm C (chemotherapy) using the frequentist methods with the following formula^7^: log*HR*_AB_ = log *HR*_AC_˗log *HR*_BC_, and its standard error (SE) for the log *HR* was $SE\;\;\left(\log HR\mathrm{AB}\right)=\sqrt{SE{\left(\log HR\mathrm{AC}\right)}^{2}+SE{\left(\log HR\mathrm{BC}\right)}^{2}}$. RR was evaluated similarly using this formula.

All statistical analyses were conducted using STATA (version 12.0). A two‐sided *P* of <.05 defined statistical significance.

## 3 RESULTS

### 3.1 Characteristics of the eligible studies

**FIGURE 1:**
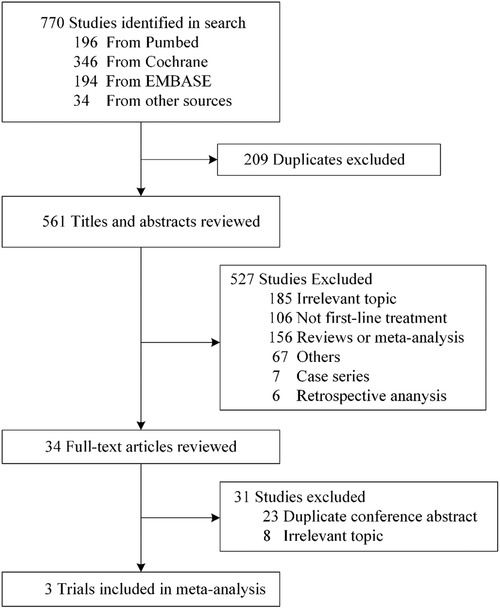
Flow diagram of trial selection

Three studies including 2372 patients fulfilled the predefined inclusion criteria. Detailed study selection process is presented in Figure 1. Risk of bias was assessed according to the Cochrane collaboration's tool (Supporting Information Table 1) and the only source of bias was the absence of data regarding immune‐related adverse events in CheckMate 227.

The main characteristics of the included studies are shown in Table 1. One study compared N‐I with chemotherapy (CheckMate 227 part 1a). The other two studies compared PEM with chemotherapy (KEYNOTE‐024 and KEYNOTE‐042). The median follow‐up periods were 29.3, 25.2, and 12.8 months, respectively. Of the 2372 patients included, 396 were from N‐I group, 1371 from chemotherapy group, and 791 from PEM group.

**TABLE 1:** Baseline characteristics and outcomes of included trials

Items	CheckMate 227		KEYNOTE‐024		KEYNOTE‐042	
Baseline characteristics	N‐I	Chemotherapy	PEM	Chemotherapy	PEM	Chemotherapy
All eligible patients	396	397	154	151	637	637
Median age (y)	64.0	64.0	64.5	66.0	63.0	63.0
Male sex (%)	64.4	65.5	59.7	62.9	70.6	71.0
Region (%)						
East‐Asia	20.5	20.4	13.6	12.6	29.0	29.0
Non‐East Asia	79.6	79.6	86.4	87.4	71.0	71.0
ECOG^a^ score (%)						
0	34.1	33.8	35.1	35.1	31.1	30.1
1	65.7	65.2	64.3	64.9	68.9	69.9
Smoking status (%)						
Current/former	84.3	85.6	96.8	87.4	77.7	78.0
Never	14.1	12.8	3.2	12.6	22.3	22.0
Unknown	1.5	1.5	0	0	0	0
Histologic type (%)						
Squamous	29.5	29.2	18.8	17.9	38.1	39.1
Non‐squamous	70.5	70.8	81.2	82.1	61.9	60.9
PD‐L1 TPS (%)						
≥1	100.0	100.0	100.0	100.0	100.0	100.0
1‐49	48.2	51.6	0	0	53.1	52.9
≥50	51.8	48.4	100.0	100.0	46.9	47.1
PD‐L1 expression assay^b^	28‐8 pharmDx		22C3 pharmDx		22C3 pharmDx	
Interventions	N‐I^c^	AP or GP^d^	PEM^e^	AP or GP or TP^f^	PEM^e^	AP or TP^g^
Endpoints						
Follow‐up time (mo)	29.3		25.2		12.8	
PD‐L1 ≥ 1%						
OS (mo), HR (95% CI)	17.1 vs 14.9, 0.79 (0.65‐0.96)				16.7 vs 12.1, 0.81 (0.71‐0.93)	
PFS (mo), HR (95% CI)	5.1 vs 5.6, 0.82 (0.69‐0.97)				5.4 vs 6.5, 1.07 (0.94‐1.21)	
ORR (%)	36 vs 30				27 vs 27	
mDOR (mo)	23.2 vs 6.2				20.2 vs 8.3	
PD‐L1 = 1‐49%						
OS (mo), HR (95% CI)	15.1 vs 15.1, 0.94 (0.75‐1.18)				13.4 vs 12.1, 0.92 (0.77‐1.11)	
PD‐L1 ≥ 50%						
OS (mo), HR (95% CI)	21.2 vs 14.0, 0.70 (0.55‐0.90)		30.0 vs 14.2, 0.63 (0.47‐0.86)		16.7 vs 12.1, 0.69 (0.56‐0.85)	
PFS (mo), HR (95% CI)	6.7 vs 5.6, 0.62 (0.49‐0.79)		10.3 vs 6.0, 0.50 (0.37‐0.68)		7.1 vs 6.4, 0.81 (0.67‐0.99)	
ORR (%)	44 vs 35		45 vs 28		39 vs 32	
mDOR (mo)	31.8 vs 5.8		NR vs 6.3		20.2 vs 10.8	

Abbreviations: PD‐L1 TPS, PD‐L1 tumor proportion score; N‐I, nivolumab + ipilimumab; PEM, pembrolizumab; OR, overall survival; PFS, progression‐free survival; ORR, objective response rate; mDOR, median duration of response; 95% CI, 95% confidence interval (CI); mo, months.

a Performance‐status evaluation of the Eastern Cooperative Oncology Group.

b PD‐L1 expression status was determined using PD‐L1 IHC 28‐8 pharmDx assay (Code SK005) and PD‐L1 IHC 22C3 pharmDx assay (Dako North America).

c Nivolumab (3 mg/kg Q2W) + ipilimumab (1 mg/Q6W).

d AP: pemetrexed (500 mg/m2 Q3W) + cisplatin (75 mg/m2 Q3W)/carboplatin (AUC = 5‐6 Q3W); GP: gemcitabine (1000 or 1250/m2) + cisplatin (75 mg/m2) or gemcitabine (1000 mg/m2) + carboplatin (AUC = 5 Q3W).

e Pembrolizumab 200 mg Q3W.

f AP: pemetrexed (500 mg/m2 Q3W) + cisplatin (75 mg/m2 Q3W)/carboplatin (AUC = 5‐6 Q3W); GP: gemcitabine (1250 mg/m2 Q3W) + cisplatin (75 mg/m2 Q3W)/carboplatin (AUC = 5‐6 Q3W); TP: paclitaxel (200 mg/m2 Q3W) + carboplatin (AUC = 5‐6 Q3W).

g AP: pemetrexed (500 mg/m2 Q3W) + carboplatin (AUC = 5‐6 Q3W); TP: paclitaxel (200 mg/m2 Q3W) + carboplatin (AUC = 5‐6 Q3W).

### 3.2 Direct comparisons of N‐I/PEM versus chemotherapy

For patients with tumor PD‐L1 level of 1% or greater, those receiving N‐I experienced improved PFS (HR_N‐I/chemo_ 0.82, 95% CI 0.69‐0.97) and OS (HR_N‐I/chemo_ 0.79, 95% CI 0.65‐0.96) compared with those receiving chemotherapy (Table 1). Similar OS improvement was observed with PEM relative to chemotherapy (HR_PEM/chemo_ 0.81, 95% CI 0.71‐0.93). However, PEM did not result in clear PFS benefit compared with chemotherapy (HR_PEM/chemo_ 1.07, 95% CI 0.94‐1.21). Neither N‐I (RR_N‐I/chemo_ 1.20, 95% CI 0.98‐1.46) nor PEM (RR_PEM/chemo_ 1.03, 95% CI 0.86‐1.23) were associated with improved response rate.

**FIGURE 2:**
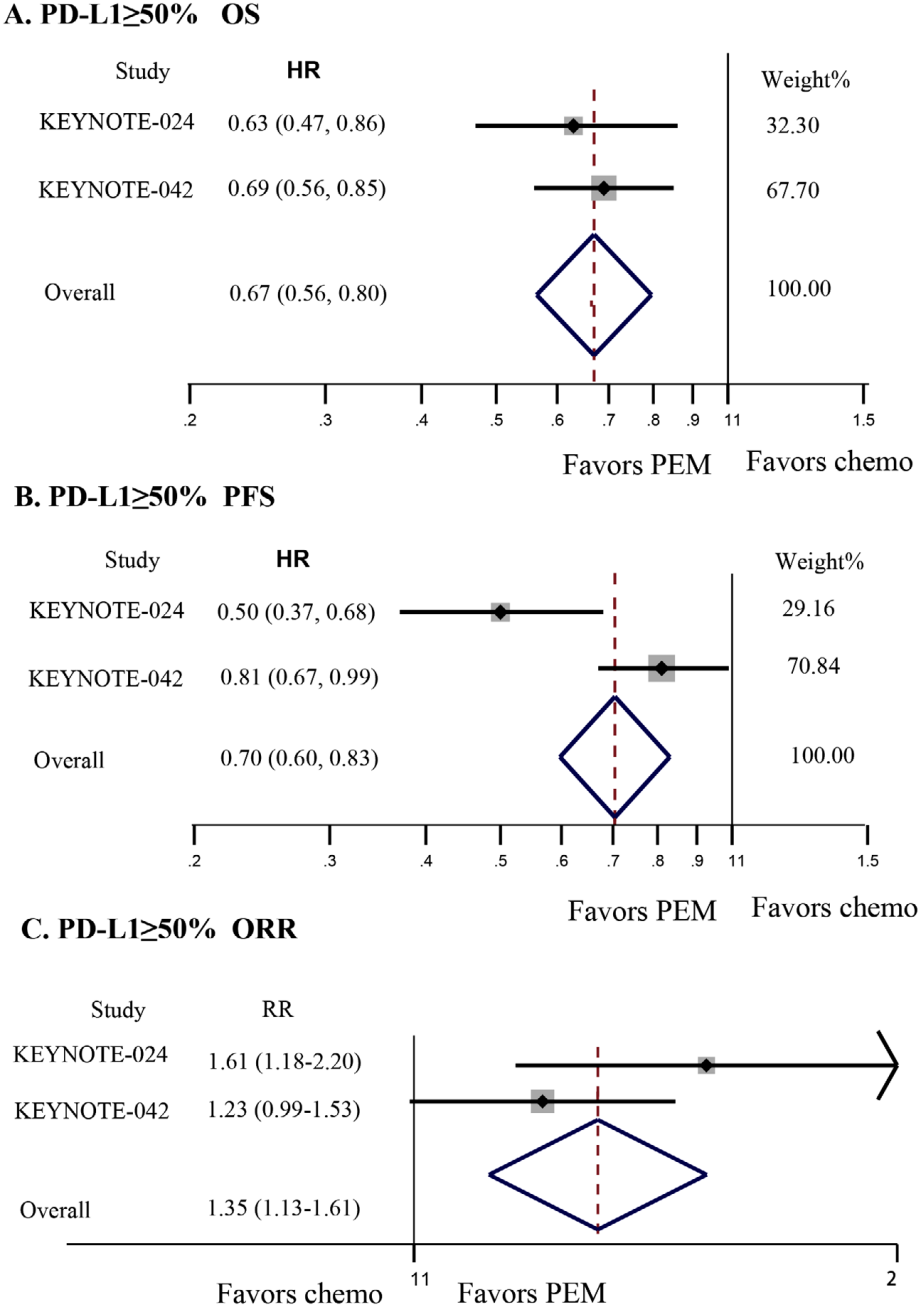
Direct comparisons between pembrolizumab (PEM) with chemotherapy (Chemo) for patients with PD‐L1 level greater than 50%. A‐C, Forest plot of hazard ratios (HRs) and risk ratio (RR) comparing overall survival (OS) (A), progression‐free survival (PFS) (B), and objective response rate (ORR) (C) between PEM with Chemo. The size of the data markers (squares) corresponds to the weight of the study in the meta‐analysis. The horizontal line crossing the square represents the 95% CI. The diamonds represent the estimated overall effect based on the meta‐analysis

Further direct analyses of benefit according to PD‐L1 level are as follows (Figure 2 and Table 2):

- ≥50%, HR_N‐I/chemo_ for death 0.70, 95% CI 0.55‐0.90; HR_PEM/chemo_ for death 0.67, 95% CI 0.56‐0.80; HR_N‐I/chemo_ for disease progression or death 0.62, 95% CI 0.49‐0.79; HR_PEM/chemo_ for disease progression or death 0.70, 95% CI 0.60‐0.83; RR_N‐I/chemo_ for response 1.16, 95% CI 0.91‐1.47; RR_PEM/chemo_ for response 1.35, 95% CI 1.13‐1.61.
- 1‐49% (only OS data available), HR_N‐I/chemo_ for death 0.94, 95% CI 0.75‐1.18; HR_PEM/chemo_ for death 0.92, 95% CI 0.77‐1.11.
- <1% (only N‐I has data), HR_N‐I/chemo_ for death 0.62, 95% CI 0.48‐0.78.

**TABLE 2:** Summary of clinical outcomes according to PD‐L1 expression level

Subgroup	N‐I versus chemo	PEM versus chemo	N‐I versus PEM
PD‐L1 ≥ 1%			
OS HR (95% CI)	0.79 (0.65‐0.96)	0.81 (0.71‐0.93)	0.98 (0.77‐1.24)
PFS HR (95% CI)	0.82 (0.69‐0.97)	1.07 (0.94‐1.21)	0.77 (0.62‐0.95)
ORR RR (95% CI)	1.20 (0.98‐1.46)	1.03 (0.86‐1.23)	1.17 (0.89‐1.52)
PD‐L1 = 1‐49%			
OS HR (95% CI)	0.94 (0.75‐1.18)	0.92 (0.77‐1.11)	1.02 (0.76‐1.37)
PD‐L1 ≥ 50%			
OS HR (95% CI)	0.70 (0.55‐0.90)	0.63 (0.47‐0.86)	1.04 (0.77‐1.42)
PFS HR (95% CI)	0.62 (0.49‐0.79)	0.50 (0.37‐0.68)	0.88 (0.66‐1.18)
ORR RR (95% CI)	1.16 (0.91‐1.47)	1.35 (1.13‐1.61)	0.86 (0.64‐1.16)
PD‐L1 < 1%			
OS HR (95% CI)	0.62 (0.48‐0.78)	No available data	

Abbreviations: PD‐L1 TPS, programmed cell death‐ligand 1 tumor proportion score; N‐I, nivolumab + ipilimumab; PEM, pembrolizumab; chemo, chemotherapy; HR, hazard ratio; 95% CI, 95% confidence interval.

### 3.3 Indirect comparisons between N‐I versus PEM of efficacy and safety

**FIGURE 3:**
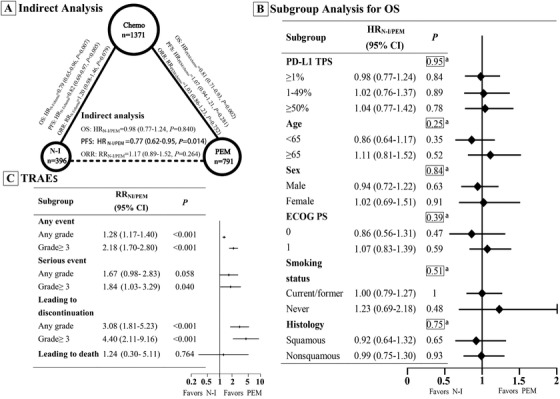
Indirect comparisons of efficacy and safety between nivolumab plus ipilimumab (N‐I) versus pembrolizumab (PEM) for patients with positive programmed cell death‐ligand 1 (PD‐L1) expression. A, Results of indirect analysis for overall survival (OS), progression‐free survival (PFS) and objective response rate (ORR) between N‐I and PEM. The solid lines represent the existence of direct comparisons between the treatments, whereas the dashed line represents the indirect comparison between N‐I versus PEM. The size of the circle corresponds to the number of enrolled patients. B, Forest plot of hazard ratios (HRs) for OS in all subgroups between N‐I and PEM. *P‐*value with a marker^a^ demonstrates the significance of differences between the subgroups. C, Forest plot of risk ratios (RRs) for treatment‐related adverse events (TRAEs) between N‐I and PEM. The horizontal line crossing the square represents the 95% confidence interval (CI) in (B) and (C). The diamonds represent the estimated overall effect based on the meta‐analysis. All statistical tests were two‐sided. Abbreviations: chemo, chemotherapy

Results from indirect comparisons showed that N‐I was statistically associated with longer PFS than PEM (HR_N‐I/PEM_ 0.77, 95% CI 0.62‐0.95). However, N‐I was not superior to PEM in terms of OS (HR_N‐I/PEM_ 0.98, 95% CI 0.77‐1.24) and ORR (RR_N‐I/PEM_ 1.17, 95% CI 0.89‐1.52) (Figure 3A). In subgroup analyses, OS was comparable between N‐I and PEM in pre‐specified subgroups including PD‐L1 level, gender, smoking status, Eastern Cooperative Oncology Group performance status (PS), and histology (Figure 3B). Nevertheless, there was a trend toward improved OS with N‐I versus PEM in younger patients (<65 years: HR_N‐I/PEM_ 0.86, 95% CI 0.64‐1.17) and patients of good PS (PS 0: HR_N‐I/PEM_ 0.86, 95% CI 0.56‐1.31).

Analyses of TRAEs suggested a less‐favorable toxicity profile with N‐I relative to PEM (Figure 3C). The rate of all grades (RR 1.28, 95% CI 1.17‐1.40) and ≥grade 3 (RR 2.18, 95% CI 1.7‐2.8) TRAEs were both significantly higher with N‐I compared with PEM. The rate of TRAEs leading to drug discontinuation occurred more frequently in those receiving N‐I (RR 3.08, 95% CI 1.81‐5.23). Treatment‐related deaths were similar between N‐I and PEM (RR 1.24, 95% CI 0.30‐5.11). Risks of some commonly reported TRAEs are presented in Table 3.

**TABLE 3:** Relative risks for treatment‐related adverse events with N‐I versus PEM

		Relative risk for N‐I versus PEM		
Treatment‐related adverse events		RRs	95% CI (*P*)	logSE
Rash	Any grade	1.91	1.00‐3.62 (.049)	0.328
	Grade ≥ 3	2.78	0.05‐168.18 (.625)	2.093
Diarrhea	Any grade	2.33	1.41‐3.87 (.001)	0.259
	Grade ≥ 3	0.83	0.11‐6.41 (.862)	1.041
Pruritus	Any grade	5.17	1.63‐16.39 (.005)	0.588
	Grade ≥ 3	1.02	0.01‐74.71 (.992)	2.189
Fatigue	Any grade	1.70	1.12‐2.58 (.013)	0.213
	Grade ≥ 3	5.31	1.10‐25.56 (.037)	0.802
Decreased appetite	Any grade	2.04	1.31‐3.16 (.002)	0.224
	Grade ≥ 3	2.51	0.46‐13.67 (.288)	0.865
Asthenia	Any grade	2.01	1.10‐3.68 (.023)	0.308
	Grade ≥ 3	4.27	0.68‐26.7 (.121)	0.936
Nausea	Any grade	1.60	1.04‐2.47 (.032)	0.22
	Grade ≥ 3	3.22	0.24‐42.85 (.377)	1.321
Vomiting	Any grade	2.36	1.18‐4.7 (.015)	0.352
	Grade ≥ 3	0.24	0.01‐3.88 (.314)	1.424
Constipation	Any grade	1.80	0.83‐3.89 (.135)	0.394
	Grade ≥ 3			
Anemia	Any grade	0.79	0.43‐1.45 (.445)	0.313
	Grade ≥ 3	1.95	0.59‐6.44 (.272)	0.609
Neutrophil count decreased	Any grade	3.57	0.74‐17.29 (.115)	0.805
	Grade ≥ 3	1.13	0.03‐36.79 (.947)	1.779
Neutropenia	Any grade	0.31	0.04‐2.65 (.286)	1.09
	Grade ≥ 3	0.68	0.03‐16.77 (.815)	1.634

Abbreviations: N‐I, nivolumab plus ipilimumab; PEM, pembrolizumab.

## 4 DISCUSSION

To the best of our knowledge, this is the first study to compare the efficacy and safety between N‐I and PEM in NSCLC, via indirect comparison. This hypothesis‐generating study revealed that nivolumab plus low‐dose, long‐interval ipilimumab had superior PFS over PEM as 1L treatment for patients with PD‐L1‐positive advanced NSCLC. However, this benefit was absent in terms of OS (across different subgroups) and ORR. Overall, patients receiving N‐I experienced more TRAEs than those receiving PEM.

The KEYNOTE‐042 was the pivotal study showing that PEM outperformed chemotherapy as 1L treatment of PD‐L1‐positive advanced NSCLC. However, exploratory analysis implied that PEM provided long‐term survival to only those with PD‐L1 TPS of ≥50% but not those between 1% and 49%. Furthermore, the CheckMate 026 study showed that neither PFS nor OS were prolonged with nivolumab in patients with >5% tumor PD‐L1 staining.^8^ These data implied that monotherapy blockade of PD‐1 failed to provide benefit for a broader population of patients. One explanation is that PD‐1/PD‐L1 engagement is not the only mediator for immune evasion of tumor cells. Among this process, CTLA‐4 plays an important role in the early‐phase regulation of T‐cell proliferation, whereas PD‐1 participates in the latter phase. This complementary mechanism of action makes the dual inhibition of CTLA‐4 and PD‐1 an appealing approach,^9^ which has been clinically proved in melanoma^10^ and renal cell carcinoma.^11^

Unexpectedly, our analysis showed comparable efficacy between N‐I and PEM in NSCLC except that the latter one was associated with longer PFS. The rationale for the absence of OS benefits remains to be unveiled. Possible explanations may include insufficient synergy of dual inhibition of CTLA‐4 and PD‐1 in NSCLC, limited efficacy of CTLA‐4 blockade in NSCLC, inappropriate dosing and interval of ipilimumab, lack of established predictive biomarkers (given that both PD‐L1 and tumor mutation burden failed), unbalanced post‐progression treatment, and unequal performance of chemotherapy arms across different studies. An ongoing phase III study is evaluating PEM plus ipilimumab versus PEM in NSCLC patients with PD‐L1 TPS of ≥50% (KEYNOTE‐598, NCT03302234), which will further provide answers for whether there is an added value of ipilimumab to PD‐1 blockade and whether there is a difference between nivolumab and PEM, when combined with ipilimumab.

Noteworthy, exploratory analysis indicates that N‐I improves OS in patients with PD‐L1‐negative NSCLC. This is clinically relevant because chemotherapy is currently unavoidable in this subset of patients. Additionally, in our subgroup analyses, a trend toward improved OS was observed in younger patients and in patients with good performance status who were receiving N‐I therapy, which required further investigation. With increasing studies exploring the frontline immunotherapy of NSCLC, there will be growing challenges to determine which treatment is best for patients with different clinicopathological characteristics: chemotherapy plus immunotherapy, immunotherapy alone, immunotherapy plus immunotherapy, or immunotherapy plus anything else.

Several limitations should be considered. First, this is an integrated analysis of published results rather than individual patient's data. Second, we lack head‐to‐head comparisons. In addition, only three qualified trials were included. Therefore, the interpretation of the results needs additional caution. Considering these limitations, head‐to‐head randomized trials will be required to directly compare PEM against N‐I.

In conclusion, our study indicates that N‐I and PEM provide comparable overall survival benefit for PD‐L1‐positive NSCLC, though mostly driven by the group of PD‐L1 ≥ 50%. N‐I was associated with superior PFS relative to PEM but at meaningful cost of toxicities. Both regimens spare patients from 1L chemotherapy and change the practice paradigm of NSCLC. Clinicians should carefully balance the efficacy, toxicity, and costs of different regimens in order to optimize clinical outcomes.

## AVAILABILITY OF DATA AND MATERIALS

All the data generated or analyzed during this study are included in the published article.

## AUTHOR CONTRIBUTIONS

Y.X.Z., Y.Q.Z., and G.F.G. contributed to data acquisition, data interpretation, and statistical analysis and drafting of the manuscript. X.Y.C., H.Y., and Y.Y.C. contributed to data acquisition, data interpretation, and statistical analysis. B.Z., S.D.H., and L.Z. contributed to the study design, data acquisition, data interpretation, and statistical analysis. All the authors contributed to critical revision of the manuscript.

## CONFLICT OF INTEREST

The authors declare that they have no conflict of interest.

## FUNDING INFORMATION

This study was funded by grants 2016YFC0905500 and 2016YFC0905503 from the National Key R&D Program of China; 81903176, 81972898, 81602005, 81702283, 81872499, and 81602011 from the National Natural Science Funds of China; 16zxyc04 from the Outstanding Young Talents Program of Sun Yat‐sen University Cancer Center; 17ykpy81 from the Central Basic Scientific Research Fund for Colleges‐Young Teacher Training Program of Sun Yat‐sen University; 2019A1515011596, 2017B020227001 from the Science and Technology Program of Guangdong Province. The funding sources had no role in the design and conduct of the study; collection, management, analysis, and interpretation of the data; preparation, review, or approval of the manuscript; and decision to submit the manuscript for publication.

## Supporting information

Supporting Information
