# Supplementary material for: Nivolumab plus ipilimumab versus pembrolizumab as chemotherapy‐free, first‐line treatment for PD‐L1‐positive non‐small cell lung cancer
Source: Clin Transl Med. 2020 Apr 7;10(1):107–15. doi: 10.1002/ctm2.14 (PMC7240850; doi:10.1002/ctm2.14)
Supplement: Supplementary file 1 — Supporting Information [file CTM2-10-107-s001.pdf]

Supplemental Method

Search strategies for PubMed, EMBASE, and Cochrane database

Pubmed:195 results

((((((((((((((("Carcinoma, Non-Small-Cell Lung"[Mesh]) OR "Carcinoma, Non Small Cell Lung"[Title/Abstract]) OR "Carcinomas, Non-Small-Cell Lung"[Title/Abstract]) OR "Lung Carcinoma, Non-Small-Cell"[Title/Abstract]) OR "Lung Carcinomas, Non-Small-Cell"[Title/Abstract]) OR "Non-Small-Cell Lung Carcinomas"[Title/Abstract]) OR "Nonsmall Cell Lung Cancer"[Title/Abstract]) OR "Non-Small-Cell Lung Carcinoma"[Title/Abstract]) OR "Non Small Cell Lung Carcinoma"[Title/Abstract]) OR "Carcinoma, Non-Small Cell Lung"[Title/Abstract]) OR "Non-Small Cell Lung Cancer"[Title/Abstract]) OR "Non Small Cell Lung Cancer"[Title/Abstract]) OR "NSCLC"[Title/Abstract]))) AND ((((((((((((((("Nivolumab"[Mesh]) OR "Nivolumab"[Title/Abstract]) OR "Opdivo"[Title/Abstract]) OR "ONO-4538"[Title/Abstract]) OR "ONO 4538"[Title/Abstract]) OR "ONO4538"[Title/Abstract]) OR "MDX-1106"[Title/Abstract]) OR "MDX 1106"[Title/Abstract]) OR "MDX1106"[Title/Abstract]) OR "BMS-936558"[Title/Abstract]) OR "BMS 936558"[Title/Abstract]) OR "BMS936558"[Title/Abstract])) AND (((((((((("Ipilimumab"[Mesh]) OR "Ipilimumab"[Title/Abstract]) OR "Anti-CTLA-4 MAb Ipilimumab"[Title/Abstract]) OR "Anti CTLA 4 MAb Ipilimumab"[Title/Abstract]) OR "Ipilimumab, Anti-CTLA-4 MAb"[Title/Abstract]) OR "Yervoy"[Title/Abstract]) OR "MDX 010"[Title/Abstract]) OR "MDX010"[Title/Abstract]) OR "MDX-010"[Title/Abstract]) OR "MDX-CTLA-4"[Title/Abstract]) OR "MDX CTLA 4"[Title/Abstract]))) OR ((((("pembrolizumab" [Supplementary Concept]) OR "pembrolizumab"[Title/Abstract]) OR "Keytruda"[Title/Abstract]) OR "MK-3475"[Title/Abstract]) OR "lambrolizumab"[Title/Abstract]))) AND ((((((("Randomized Controlled Trials as Topic"[Mesh]) OR "Clinical Trials, Randomized"[Title/Abstract]) OR "Trials, Randomized Clinical"[Title/Abstract]) OR "Controlled Clinical Trials, Randomized"[Title/Abstract]) OR "Clinical Trials"[Title/Abstract]) OR "Clinical

Trial"[Title/Abstract]) OR "Randomized Controlled Trials"[Title/Abstract]))

Embase:194 results

('non small cell lung cancer'/exp OR 'non small cell lung cancer':ab,ti OR 'pulmonary non small cell carcinoma':ab,ti OR 'pulmonary non small cell cancer':ab,ti OR 'non small cell pulmonary carcinoma':ab,ti OR 'non small cell pulmonary cancer':ab,ti OR 'non small cell lung carcinoma':ab,ti OR 'non small cell cancer, lung':ab,ti OR 'non small cell bronchial cancer':ab,ti OR 'lung non small cell carcinoma':ab,ti OR 'lung non small cell cancer':ab,ti OR 'lung cancer, non small cell':ab,ti OR 'carcinoma, non-small-cell lung':ab,ti OR 'bronchial non small cell carcinoma':ab,ti OR 'bronchial non small cell cancer':ab,ti OR 'NSCLC':ab,ti) AND ('pembrolizumab'/exp OR 'pembrolizumab':ab,ti OR 'lambrolizumab':ab,ti OR 'mk 3475':ab,ti OR 'mk3475':ab,ti OR 'sch 900475':ab,ti OR 'sch900475':ab,ti OR 'keytruda':ab,ti OR (('nivolumab'/exp OR 'nivolumab':ab,ti OR 'bms 936558':ab,ti OR 'bms936558':ab,ti OR 'cmab 819':ab,ti OR 'cmab819':ab,ti OR 'mdx 1106':ab,ti OR 'mdx1106':ab,ti OR 'ono 4538':ab,ti OR 'ono4538':ab,ti OR 'opdivo':ab,ti) AND ('ipilimumab'/exp OR 'ipilimumab':ab,ti OR 'bms 734016':ab,ti OR 'bms734016':ab,ti OR 'mdx 010':ab,ti OR 'mdx 101':ab,ti OR 'mdx010':ab,ti OR 'mdx101':ab,ti OR 'strentarga':ab,ti OR 'yervoy':ab,ti))) AND ('randomized controlled trial'/exp OR 'randomized controlled trial':ab,ti OR 'controlled trial, randomized':ab,ti OR 'randomised controlled study':ab,ti OR 'randomised controlled trial':ab,ti OR 'randomized controlled study':ab,ti OR 'trial, randomized controlled':ab,ti OR 'controlled clinical trial':ab,ti)

Cochrane:346 results (337 trials, 7 protocols, 2 reviews)

#1 MeSH descriptor: [Carcinoma, Non-Small-Cell Lung] explode all trees

#2 “lung” AND ("Non Small Cell" OR "Non-Small Cell" OR "Non-Small-Cell") OR "NSCLC"

#3 #1 OR #2

#4 MeSH descriptor: [Nivolumab] explode all trees

#5 "Nivolumab" OR "MDX 1106" OR "MDX1106" OR "MDX-1106" OR "ONO-4538" OR "ONO 4538" OR "ONO4538" OR "BMS 936558" OR "BMS-936558" OR

"BMS936558" OR "Opdivo"

#6 #4 OR #5

#7 MeSH descriptor: [Ipilimumab] explode all trees

#8 "Ipilimumab" OR "MDX-010" OR "MDX010" OR "MDX 010" OR "Anti CTLA 4 MAb Ipilimumab" OR "Ipilimumab, Anti-CTLA-4 MAb" OR "Anti-CTLA-4 MAb Ipilimumab " OR "Yervoy" OR "MDX-CTLA-4" OR "MDX CTLA 4"

#9 #7 OR #8

#10 #6 AND #9

#11 "Pembrolizumab"

#12 "Pembrolizumab" OR "Lambrolizumab" OR "Keytruda" OR "MK-3475"

#13 #11 OR #12

#14 MeSH descriptor: [Randomized Controlled Trial] explode all trees

#15 "Randomized Controlled Trial" OR "Controlled Clinical Trials, Randomized" OR "Clinical Trials, Randomized" OR "Trials, Randomized Clinical" OR "Clinical trial" OR "Clinical trials"

#16 #14 OR #15

#17 #10 OR #13

#18 #3 AND #17 AND #16

Supplemental Table 1. Quality assessment by Cochrane Collaboration's tool.

Trial	Sequence generation	Allocation concealment	Blinding	Incomplete outcome data	Selective reporting	Other source of bias
KEYNOTE024	Adequate	Adequate (Central allocation)	Adequate [a]	Adequate	Adequate	
KEYNOTE042	Adequate	Adequate (Central allocation)	Adequate [a]	Adequate	Adequate	
CheckMate227	Adequate	Adequate (Central allocation)	Adequate [a]	Adequate	Adequate	**Inadequate data** [b]

[a]The sponsor, investigator and subject were aware of the treatment administration but the response to treatment was assessed by means of blinded, independent, central radiologic review.

[b]Absence of immune-related adverse events.
